# Micromechanical interlocking structure at the filler/resin interface for dental composites: a review

**DOI:** 10.1038/s41368-023-00226-3

**Published:** 2023-05-31

**Authors:** Shuning Zhang, Xiao Wang, Jiawei Yang, Hongyan Chen, Xinquan Jiang

**Affiliations:** Department of Prosthodontics, Shanghai Ninth People’s Hospital, Shanghai Jiao Tong University School of Medicine; College of Stomatology, Shanghai Jiao Tong University; Shanghai Engineering Research Center of Advanced Dental Technology and Materials; National Center for Stomatology; National Clinical Research Center for Oral Diseases; Shanghai Key Laboratory of Stomatology; Shanghai Research Institute of Stomatology, Shanghai, China

**Keywords:** Composite resin, Dental biomaterials

## Abstract

Dental resin composites (DRCs) are popular materials for repairing caries or dental defect, requiring excellent properties to cope with the complex oral environment. Filler/resin interface interaction has a significant impact on the physicochemical/biological properties and service life of DRCs. Various chemical and physical modification methods on filler/resin interface have been introduced and studied, and the physical micromechanical interlocking caused by the modification of fillers morphology and structure is a promising method. This paper firstly introduces the composition and development of DRCs, then reviews the chemical and physical modification methods of the filler/resin interface, mainly discusses the interface micromechanical interlocking structures and their enhancement mechanism for DRCs, finally give a summary on the existing problems and development potential.

## Introduction

Oral diseases link to diabetes, cardiovascular disease, and respiratory disease, giving a significantly negative impact on human health and quality of life.^1,2^ Among all kinds of oral diseases, caries has the highest incidence, and can cause many other diseases.^3–6^ Repair materials play an extremely important role in caries treatment.^2^ Since the 1950s, dental resin composites (DRCs) were introduced as the filling treatment of caries, and have been developed considerably due to their excellent simulated esthetics, biocompatibility, suitable mechanical properties, and easy clinical operability.^7–9^

Various types of DRCs have been developed based on different standards, which can be divided into flowable composites,^10^ packable composites,^11^ “bulk-fill” composites,^12^ polymer-infiltrated ceramic network (PICN),^13^ self-adhesive composites,^14^ depending on the clinical applications (Fig. S1, Supplementary information). Although each DRC has its own characteristics, there are still restorative fractures or secondary caries during clinical application, mainly due to the poor mechanical performance and functionality.^15–18^ Various approaches have been employed to relieve these problems,^19,20^ and optimizing the interface interaction between inorganic (fillers) and organic (resin matrix) is one of the most effective methods.^21^ According to summarizing the existing research on DRCs, the improved filler/resin interfacial interaction is mainly achieved by modifying the fillers, which further improves the comprehensive properties of DRCs. Among all the methods to improve the interface interaction, micromechanical interlocking is a simple and effective physical method, which is achieved by constructing the structural bonding between two phases through the infiltration or embedding on the micron or nanometer scale, making the resulting composite resistant to loading.^22–24^

In this paper, we emphatically review the chemical and physical modification technologies used to improve the filler/resin interface interaction, and comprehensively discuss the physical micromechanical interlocking structures on the interface caused by the morphology structure of fillers.

## Compositions of dental resin composites

Dental resin composites, as the name indicates, are a mixture of organic resin matrix (the mixture of resin monomers and initiators), modified inorganic fillers, as well as a small amount of dye. After mixing the resin with the fillers thoroughly, initiators can trigger the polymerization of DRCs in situ or in vitro for direct or indirect repair.^8^

### Resin matrix

The resin matrix is a polymer network formed by the polymerization of organic monomers under the action of initiators, which can further wrap inorganic fillers to form a whole together. Commonly used resin monomers are methacrylic-based resins, such as bisphenol A glycerolate dimethacrylate (Bis-GMA), triethylene glycol dimethacrylate (TEGDMA), and urethane dimethacrylate (UDMA), and so on.^25^ The polymerization process of the monomers results in the volume shrinkage of corresponding DRCs, and the polymerization is rarely complete because of the trapped radicals in the cross-linked networks, all of these are harmful to the properties of DRCs and unconducive to their clinical application.^26,27^

Many new monomers with low polymerization shrinkage and high degree of conversion of C = C bonds should be developed.^28–31^ In addition, the composition and ratio of resin monomers, as well as the more efficient photoinitiation system still need to be optimized to finally find a balance in viscosity, mechanical properties, polymerization shrinkage, and other properties of DRCs.

### Inorganic fillers

Fillers are the main component of currently DRCs (60%–85%), which can effectively improve the mechanical properties, reduce the polymerization shrinkage, decrease the coefficient of thermal expansion, and provide X-ray blocking of DRCs.^32^ Silica-based particles are the most commonly used inorganic filler for DRCs due to their matched refractive index with resin matrix, good stability, and well mechanical properties, etc.^8^ Other inorganic particles with their own function, such as alkaline glass, metal oxides, and hydroxyapatite (HAp), and so on are also used as fillers for DRCs.^33^ The composition, size, shape, morphology, distribution, and loading of fillers, and their bonding strength with resin matrix are the most active and significant change factors to improve the properties of DRCs.^25^

## Modification of filler/resin interface interaction

DRCs are typical organic/inorganic composites, and the filler/resin interface interaction is weak because of the organic resin matrix usually showing hydrophobicity and the inorganic filler usually presenting hydrophilic. Many researchers confirm that the filler/resin interface interaction has a significant influence on the comprehensive properties and service life of DRCs, and some scholars regard it as the third phase in composites.^21,34^ The filler/resin interface interaction can be optimized by adjusting the hydrophobic-hydrophilic property or the morphology structures of fillers, and the details are as follows.

### Chemical modification of fillers

Chemical modification of fillers mainly involves grafting the amphipathic coupling agent or polymer onto filler surface, which can form chemical bonds, such as covalent or ionic bonds, van der Waals forces, ionic interactions, and hydrogen bonds, at the filler/resin interface.^35^ This method effectively improves the interface bonding and facilitates transferring the stress from the low-modulus resin matrix to high-modulus fillers, which can prevent crack extension along the interface and thus improve the comprehensive properties of DRCs.

#### Coupling agent modification

**Fig. 1:**
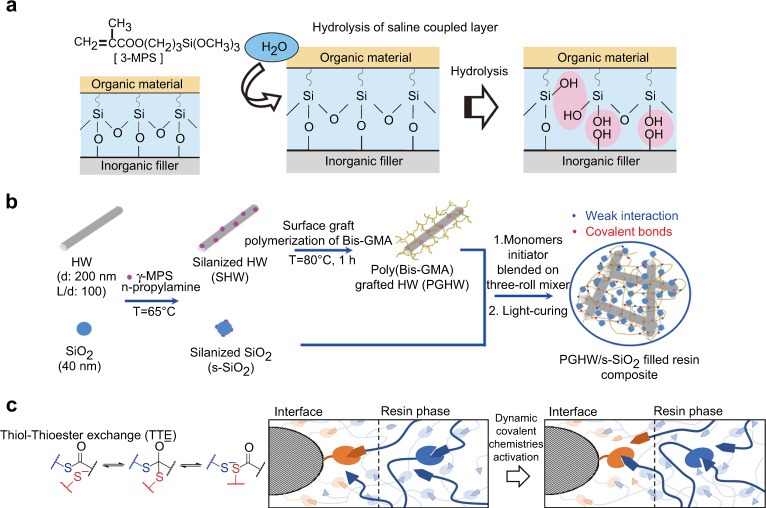
Chemical modification of fillers. **a** The principle of γ-MPS modified filler surface and the hydrolysis of the formed filler/resin interface.^37^ **b** Synthesis of poly (Bis-GMA)-grafted silanized hydroxyapatite whiskers (PGSHW) and preparation of composite with PGSHW and silanized SiO_2_ fillers.^57^ **c** The mechanism of thiol-thioester exchange (TTE) and its relaxation of stress at the resin/ filler interface, the red and blue arrows represent the free thiol and thioester linkage, and the arrows colors is correspond to the colors of the chemical bonds on the left.^62^ Figures adapted with permission from refs. ^37,57,62^

Coupling agent modification of fillers is the most commonly used method to improve the filler/resin matrix interface interaction by forming a chemical bonding between them. It forms a molecular bridge by connecting the fillers with its terminal hydrophilic group and polymerizing the resin matrix with its hydrophobic group.^36^ The silane coupling agent, which can be expressed as Y-(CH_2_)_k_-Si-X_3_, is the most commonly used coupling agent,^37^ and 3-Methacrylamidopropyltrimethoxysilane (γ-MPS) is popular in DRCs (Fig. 1a). The suitable concentration of silane coupling agent that grafts onto filler surface is important to the filler/resin interface interaction.^29,38,39^ It should be noted that, the formed Si-O-Si bond at the interface is prone to hydrolysis in humid environment, and there must be active sites on filler surface.^21^ Therefore, the coupling agents with strong hydrophobicity or with the non-linking function were studied.^40,41^

Titanium-derived coupling agents (TCA) and zirconate coupling agents have been also used for filler modification for composites.^42–45^ TCA can react with free protons on the filler surface forming an organotitanium monomolecular layer to combine with organic,^46,47^ and can reduce the surface energy of fillers.^43,46^ Zirconate coupling agents can form hydrogen bonds with fillers and cross-link with the resin by establishing donor-acceptor interactions, which is beneficial to improving the shear bond strength and hydrolytic stability of the interface, and therefore improve the properties of composites.^48,49^ However, there are few studies on the use of these two types of coupling agents for DRCs because of their tedious operation and high production cost.

#### Polymer modification

Polymer grafting onto the filler surface is another chemical modification method to improve the filler/resin interface interaction (Fig. 1b). This method allows filler show hydrophobicity, which makes them easier to disperse in the resin matrix.^50–54^ Besides, the grafted polymers can act as a functional transition layer and interact with the organic monomers, thereby enhancing the filler/resin interface bonding.^55,56^ Various polymers have been grafted onto filler surface, such as PMMA, Bis-GMA, polyacrylic acid, MA-POSS, and oligomers, and the results demonstrated that the grafting polymer layers and their content had significant effects on the mechanical properties, polymerization shrinkage, absorption-solubility properties, degree of conversion, as well as other properties of DRCs.^55–60^ However, chemical grafting involves complex operational steps so is not simple to implement.

#### Dynamic covalent bonding

The dynamic covalent chemistry (DCC) was also introduced at the filler/resin interface to relieve the microcracks and the low fracture toughness of methacrylate-based composites, further enhancing the properties of DRCs. This process enables dynamic bond exchange in the polymer backbone while conserving the overall covalently bonded structure (Fig. 1c),^61^ which represents a fundamental shift by relaxing interfacial stresses and further enhance the properties of DRCs. The covalent adaptable networks such as those based on reversible addition-fragmentation chain transfer (RAFT) and the thiol-thioester exchange (TTE) have been successfully implemented in dense networks.^61,62^ The introduce of TTE reaction at the resin-filler interface in conventional DRCs leaded to their significant improvement performance including 30% reduction in polymerization stress, 60% improvement in flexural modulus (FM), and 40% improvement in the flexural strength (FS).^62^ There have few studies using this method and it may be of significant clinical value for extending the lifetime of dental restorations.

### Physical modification of fillers

As discussed above, there are some problems with chemical modification of fillers to improve the filler/resin interface for DRCs. For these, the physical modification methods are studied, which mainly involves adjusting the morphology and structure of fillers to form the micromechanical interlocking structure between fillers and resin matrix, and the fillers with various porous structures and whisker morphologies will be highlighted. Examples of the classification of micromechanical interlocking structures in DRCs and their effects on DRCs are summarized in Table S1 (Supplementary information) and the detailed discussion will be reviewed in “Micromechanical interlocking structure on filler/resin interface”.

## Micromechanical interlocking structure on filler/resin interface

Mechanical interlocking structure between different phases is benefited to forming the strong interface interaction, and thus transfer and dissipate the stress according to the hooks, friction, and leverage generated by the physical action.^63–65^ This structure, which is similar to the “brick-and-mortar” structure, is very common in nature, such as the abundant dendritic root system fully embedded in the surrounding soil, the interlocking between the “hook” and “ring” in burdock seeds, the wing-to-body locking device in beetles, as well as the shell structure in the enamel dentin boundary, the acid etching and the mixed layers during dental filling restoration, and so on.^66–69^ At the micro level, the mechanical interlocking structure exists in the interface of various composites, which is achieved by constructing the structural bonding between two phases through the infiltration or embedding on the micron or nanometer scale, naming as the micromechanical interlocking structure in this review.

For DRCs, filler with a special three-dimensional structure (porous structure or three-dimensional whisker structure) can be embedded in the resin matrix more uniformly, which is conducive to increasing the contact area between the filler and the resin matrix, so as to further form a micromechanical interlocking structure during the curing process. This can improve the interfacial interaction, dissipate the displacement between the two phases under stress in the application and improve the comprehensive properties of DRCs. Therefore, the morphological structure of the filler is crucial for the construction of micromechanical interlocking at filler/the resin matrix interface, which determines the degree of interlocking of the filler/resin. However, there are few works that describe the filler/resin matrix interface micromechanical interlocking intuitively, which may be can characterized by SEM/FIB or TEM/FIB to observe the depth of the resin matrix entering the pore structure of fillers.^70,71^ In addition, the physical micromechanical interlocking structure at the interface can avoid the problem of interfacial chemical bond hydrolysis caused by chemical modification of fillers (“Chemical modification of fillers”), which is of great significance and value for the application of DRCs in oral environment.

Based on this, some researchers have roughened the filler surface by surface fusion, deposition of small particles^72,73^ or selective metal etching,^74^ which can increase the interfacial contact area and enhance the friction and energy of filler pulled out of the resin matrix. However, it should be noted that these methods are only auxiliary reinforcement methods, which are different from the interlocking structure. This review focuses on the micromechanical interlocking formed by inorganic fillers with well-defined morphological structures, and these fillers can be specifically divided into two categories: porous structure fillers (surface pore, interconnected pore, porous scaffold, porous nanocluster) that allow resin matrix to penetrate their porous channels and three-dimensional whisker structure fillers that embedded in resins, which will be discussed in detail in the following.

### Fillers with porous morphology structure

Porous fillers, also known as inorganic phase with cavities, channels, or interstices, have been used in DRCs since 1976 due to their unique characteristics and adjustable particle size, pore size and shape, porosity, and composition. During the process of constructing porous filler-reinforced DRCs, the resin matrix can be introduced into the porous fillers by high-pressure or vacuum technologies before polymerization and thus creating the filler/resin interface micromechanical interlocking during the curing process.^75,76^ At present, the researches on porous fillers in DRCs mainly focus on surface-porous fillers, interconnected-porous fillers, porous scaffolds, and porous nanoclusters.

#### Surface-porous fillers

**Fig. 2:**
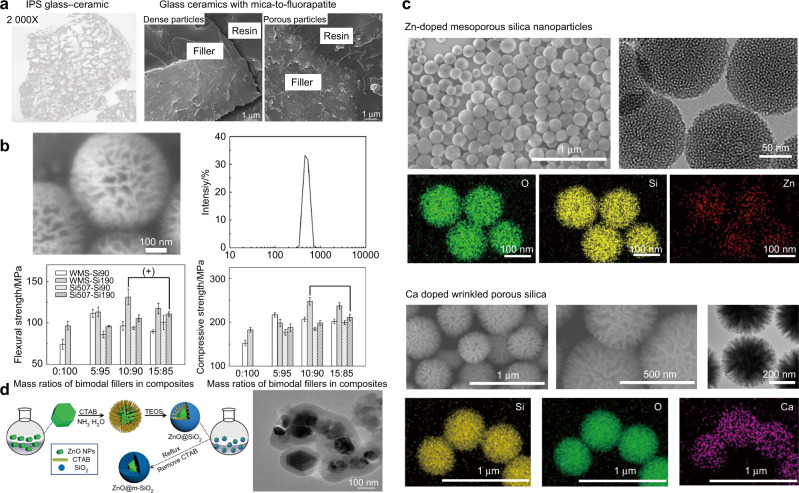
Surface-porous fillers. **a** SEM images of glass-ceramic fillers after acid etching^78^ and the fracture morphology of DRCs filled with dense particles and acid-etching porous particles.^80^ **b** SEM images and particle size distribution of WMS, and the comparison of the mechanical properties of DRCs filled with silica, WMS particles, or silica/WMS bimodal fillers.^88^ **c** SEM, TEM, and EDS mapping analysis of Zn-MSNs^91^ and Ca-WPS.^105^ **d** Synthesis and SEM images of core-shell ZnO@m-SiO_2_ particles.^90^ Figures adapted with permission from refs. ^78,80,88,90,91,105^

Inspired by the micromechanical interlocking structure, the surface-porous glass fillers formed by the etching process were first used in DRCs by Bowen and Reed.^76^ Filler with this structure has a higher surface area, which was beneficial to the stress transfer between the filler and resin matrix and improve the interface interaction. The silica filler with surface microporous structure obtained by etching can improve the enhancement effect and wear resistance of the DRCs, mainly because the microporosity structure makes the filler difficult to fall off.^77^ Glass-ceramic fillers with surface-porous structures were obtained by hydrofluoric acid etching according to the control of glass-ceramic composition and etching conditions.^77–80^ These surface-porous glass-ceramics fillers presented stronger mechanical properties by the bubble expulsion and micromechanical interlocking (Fig. 2a).

The surface-porous structure and the pore size of fillers modified by etching are uncontrollable during the etching process, which limited its application. Since 1992, the synthesis of silicate/aluminosilicate mesoporous materials using surfactants as templates has been extensively studied,^81^ and the researches of porous fillers in DRCs are mainly based on porous silica-based particles. Surfactants are used as templates to guide the formation of mesopores, and the inorganic precursors are matched with them to eventually form the corresponding porous structures. Later, it was discovered that the non-surfactant organic molecules can also act as templates,^82^ and they can direct the formation of intermediate phases through hydrogen bonds between templates and inorganic precursors in solution and/or during gelation. The particles with the mesoporous structure are obtained by the removal of templates, which have a higher surface area and controllable particle size and pore size by adjusting the concentration of the template, the type of precursor, and the mass ratio between them. During the synthetic process of porous particles, the increasing of the template concentration can result in higher surface area, larger pore volume, and pore size, and when the concentration of the template reached 30%–40%, mesopores began to dominate rather than micropores.^83,84^ Ji et al. synthesized mesoporous silica by surfactant template method, followed by synthesized poly (TMSPMA)-mesoporous silica complexes, and demonstrated that the composite exhibited higher tensile strength and toughness by mechanical property tests.^85^ The resin matrix could penetrate and polymerize within the porous structure and be confined within them forming physical cross-linking points, which also improved the mechanical properties of the composite by changing the fracture from a brittle fracture to a plastic fracture.

It should be noticed that the porous particles have a higher specific surface area and surface energy than nonporous particles, which would reduce the filler amount in composites and cannot improve their mechanical properties to the maximum extent.^86^ Praveen et al. pointed out that it is theoretically possible to increase the filler loading by reducing the surface area of porous particles, such as using different templates or high-temperature treatment of fillers caused some mesoporous collapse, or introducing nonporous particles.^87^ Wang designed monodisperse wrinkled mesoporous silica (WMS) particles with an average diameter of 496 nm and used them as fillers in DRCs (Fig. 2b).^88^ This unimodal filler had only 35% loading, but when they were co-loaded with small spherical silica particles (190 nm, Si190), the filler content of the DRCs could reach 60%, and the mechanical properties of FS, FM, compressive strength (CS), and Vickers hardness (VH) was enhanced by 17.8, 8.0, 14.3, and 29.6%, respectively.

Furthermore, using the template method, a controlled porous-shell structure can be prepared. Wang et al. synthesized the rough core-shell SiO_2_ (rSiO_2_) nanoparticles and used them as the unimodal filler in DRCs, where the mesoporous silica shell coating onto the surface of the smooth nonporous silica particle.^89^ This particle presented the surface-porous structure with controllable porous-shell thickness, and its loading mass ratio in DRCs could research to 67%. The morphology structure of this particle combined the advantages of smooth nonporous structure that improved the filler loading and the porous structure that optimized the filler/resin interface interaction, therefore improving the FS (~120 MPa), FM (~5 GPa), CS (~330 MPa), and other properties of composites.

Surface-porous fillers used in DRCs also can decrease the polymerization shrinkage and reduce the cytotoxicity of the composites,^90–92^ and these properties can be synergized with an increase in filler content.^93–95^ These results are mainly attributed to the fact that these pore structures can restrain the movement of polymer chains through the enhancement of interfacial interactions, which also reduces the release of toxic monomers such as TEGDMA and Bisphenol A, making them more suitable for dental and in vivo applications. Attik et al. investigated the cytocompatibility of the DRCs containing mesoporous filler and found that the cytotoxicity of these composites was reduced compared to that of nonporous fillers, explained by the fact that the organic components are locked in the pores and are more difficult to elute.^92^ Chiang and Bai also demonstrated that mesoporous SiO_2_ used on dental pulp cells and the DRCs containing mesoporous SiO_2_ exhibited good cytocompatibility.^91,96^

Based on the improvement of physicochemical properties and biosafety of composites through porous fillers, many studies have assigned biological activities to them.^97^ The introduction of the functional component may lead to the loss of physicochemical properties of the composite,^98^ The application of porous structures can solve such problems. And there are many examples of porous materials with high pore capacity and surface area for encapsulation and controlled release of drugs or ions, suggesting their applications can be combined with the antimicrobial or remineralization needs of dental restorations. Carpenter et al. previously loaded O^2^-protected N-diazadiylsilanes onto mesoporous silica nanoparticles, and these NO-release particles reduced the adhesion of the Streptococcus mutans.^99^ Zhang et al.^100^ fabricated the antimicrobial DRCs filled with mesoporous silica-loaded chlorhexidine (CHX) nanoparticles. The results showed that mesoporous structure still can form the filler/resin interfacial micromechanical interlocking after the addition of drugs, thus keeping the FS higher than the ISO standard. Moreover, the composite exhibited sustainable and controlled prolonged drug release properties and also provided a permanent “sponge” that could be recharged repeatedly with antimicrobial agents, which greatly facilitated the long-term maintenance of the antimicrobial properties of the dental restorative material.

ZnO shows excellent antimicrobial activity and tooth-colored characteristics, making it more suitable and interesting for DRCs.^101^ Bai et al. synthesized Zn-doped mesoporous silica nanoparticles (Zn-MSNs) by sol-gel method, where the Zn^2+^ was mixed with silica precursor and self-assembly to form antimicrobial Zn-MSNs (Fig. 2c).^91^ These particles used in DRCs can effectively avoid the problem of reducing the mechanical properties when releasing antibacterial Zn^2+^, and the 15% Zn-MSNs loading had an FS of 130 MPa and a 100% slow-release bacteriostatic effect. The core-shell structure mentioned above is another way to achieve a multifunctional filler by combining a functional core and a mesoporous morphology of the shell.^90,102^ Chen et al. designed ZnO@m-SiO_2_ core-shell structure particles and used them as fillers in DRCs (Fig. 2d). The ZnO as a nucleus to release Zn^2+^ through the open skeleton structure of mesoporous SiO_2_ shell, exhibiting up to 99.9% of antibacterial activity, and the mesoporous shells can form filler/resin interfacial micromechanical interlocking structure, improving the mechanical properties of the composite. Recently, CaF_2_/SiO_2_ core-shell nanoparticles as novel fillers for DRCs were developed, which have a good prospect of application because of their sustained fluoride ion release ability and high FS (>110 MPa).^102^

In addition to antibacterial, another major idea to address secondary caries in dental restoration is to endow it with remineralization properties. Calcium is the key ingredient for remineralization of damaged enamel and dentin.^103^ Zhang et al. and Kong et al. introduced Ca element into mesoporous silica to form functional particles, which were used as the fillers for DRCs (Fig. 2c). The composites presented excellent FM of 132 MPa by the interface micromechanical interlocking structure, and a mineralized layer can be formed on the material surface with the release of Ca^2+^, which can effectively curb the caries process.^104,105^

#### Interconnected-porous fillers

**Fig. 3:**
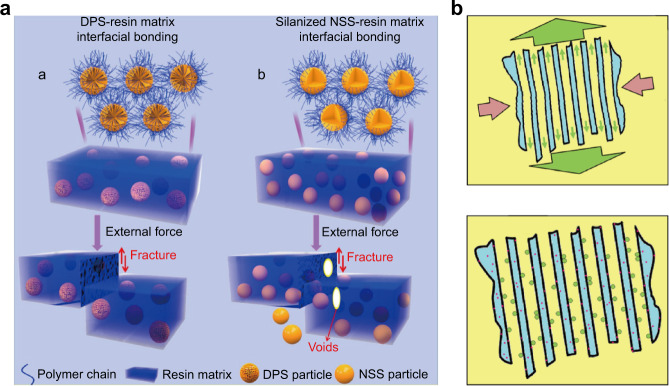
Interconnected-porous fillers. **a** The simulated morphology structure of DPS, and the schematic diagram of enhanced filler/resin interfacial bonding through the interconnected-porous structure.^106^ **b** Schematic diagram of the optimization mechanism of the interconnected-porous structure of alumina for DRCs (interconnected-porous structure can enhance interfacial interactions, allow high deformation, and insertion of bioactive particles).^70^ Figures adapted with permission from refs. ^70,106^

In addition to surface-porous structure, some researchers have also designed fillers with interconnected-porous structure and applied them to DRCs. This structure, in addition to the vertical pull-out effect and lateral force resistance, has the micromechanical interlocking effect caused by making the organic phase and inorganic phase form a closed loop respectively. Theoretically, this structure can have stronger micromechanical interlocking effect and alleviate the polymerization shrinkage of the resin matrix better than the surface-porous structure.^75^ Praveen et al. formed interconnected-porous silica particle using a non-surfactant template method, and introduced it into Bis-GMA/TEGDMA resin matrix, analyzing the mechanical strength and aging properties of the corresponding composite.^87^ The results demonstrated that the interconnected mesoporous silica fillers endowed DRCs with higher compressive strength, CM, FM, and more hydrolytic stability, but the flexural strength was only (68 ± 9) MPa, which was lower than the ISO standard.^75^ Chen et al. synthesized dendritic porous silica (DPS) with center-radial hierarchical pores and controllable particle sizes ranging from 75 to 1 000 nm according to the dynamic self-assembly (Fig. 3a).^106^ When used these DPS particles as fillers in DRCs, the DPS-500 (particle size with 500 nm) with 36% loading were uniformly and dispersedly embedded in the resin matrix, and the FS and FM were enhanced by 53.8% and 32.0% than the DRCs filled with 60% silane-treated nonporous silica with the same size. Also, similar to the surface-porous fillers, the cytocompatibility of interconnected-porous filled DRCs was also excellent.

In addition, porous alumina with interconnected-porous structure could be obtained by ball milling anodic aluminum oxide film, which was also developed as fillers for DRCs.^70^ The resin matrix could also penetrate the porous structure of the filler and form the micromechanical interlocking between filler and resin matrix interface (Fig. 3b), making the composites show better elastic modulus and aging stability than the standard composite at body temperature and 1 Hz strain frequency. This phenomenon indicated that the physical micromechanical interlocking is well resistant to the oral environment. Follow-up tests showed that this innovative composite had excellent FM and adhesion strength of dental tissues comparable to commercial composites.^107,108^ In addition, the simulations also confirmed the efficacy (stress-strength curve) of the micromechanical interlocking effect to be very similar to that of the conventional fully chemically bonded case. However, the actual obtained FS was still lower than the calculated value by finite element modeling. This discrepancy between actual measurements and theoretical simulations may be related to critical defective aspects such as bubbles, light-curing inhomogeneities, and insufficient polymerization, thus leaving great potential for improvement in the manufacturing quality of the composites.

In comparison with the spherical porous particles discussed above, the hollow structure nanotubes also have been introduced into DRCs as fillers, which also can form the micromechanical interlocking structure with the internal and external resin matrix in addition to their own enhancement for DRCs.^109^ Dafar et al. enhanced the mechanical properties of the commercially flowable DRCs according to adding TiO_2_ nanotubes (TNT). In addition, the surface modification of TNT can further enhance the mechanical properties, where the 3% methacrylic acid-functionalized nanotube-reinforced composites showed Young’s modulus of 16.8 GPa (similar to the dentin: ~17 GPa^110^), 58% greater than control composites,^111^ providing a good mechanical match to tooth. Abolfazl et al. tested the mechanical strength of DRCs filled with different content of TNT, and the results showed that low content of nanotubes (3%) had the best enhancement effect on DRCs.^112^ A small quantity and uniformly dispersed nanotubes could fully utilize the high specific surface area and tubular structure to enhance the filler/resin interface, so the stress could be transferred better in two phases. However, more nanotubes will form stress concentration areas due to agglomeration, which is not conducive to stress transfer reducing the mechanical properties of the composites.

#### Porous scaffolds

**Fig. 4:**
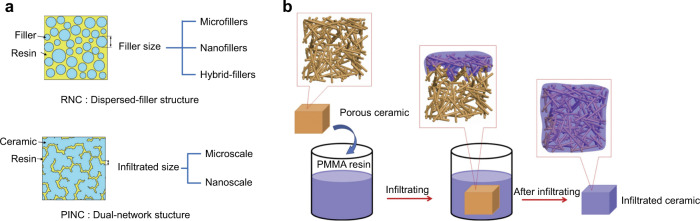
Porous scaffolds. **a** Schematic diagram of RNC and PINC.^115^ **b** Schematic diagram of polymer infiltrating porous scaffold.^118^ Figures adapted with permission from refs. ^115,118^

Indirect restorative organic-inorganic composites can be divided into resin nano-ceramics (RNC) and polymer-infiltrated ceramic-network (PICN) (Fig. 4a), and are widely used with the development of computer-aided design/computer-aided manufacturing.^113^ RNC can form a micromechanical interlocking according to the porous structure or three-dimensional whisker morphology of the fillers. PICN are made by infiltrating resin into the interconnected pores of the pre-sintered ceramics (Fig. 4b). This porous mesh structure provides a good capillary effect for the infiltration of monomers, together with vacuum and high pressure or specialized casting impregnation systems, which can create a strong interlocking between the two phases. After polymerization of the monomers, a double network interlocking structure of ceramic and polymer is formed, in which the inorganic phase accounts for 86%.^114^ The high specific gravity of this inorganic phase and the more uniform network of fully interlocking physical structures can effectively disperse stress, leading to the elasticity modulus and tensile modulus of PICN can be closer to that of dentin, while the VH is closer to that of enamel.^115,116^

The control of porosity and pore structure have an obvious impact on the construction of the micromechanical interlocking between the two phases,^117–119^ and the sintering temperature, the solid content in the suspension, and the amount of porogenic agent can control the pore volume, pore size, and pore connectivity of the porcelain block. In addition, the ceramic content is controlled to prepare PICN with different mechanical properties. The study results of Wang et al. showed that higher sintering temperature could complete the grain growth and pore shrinkage, and a moderate amount of pore-forming agent can achieve the best combination of porosity, pore connectivity, and two-phase content ratio to optimize the mechanical properties of PICN. Based on the controllable properties of inorganic porous scaffolds, Morena et al. construct the gradual and continuously aligned pores by freeze casting technique and achieve a bionic dental restorative material.^120^ Eldafrawy et al. created a ceramic density gradient by centrifugal processing and obtain a functionally graded porosity for resin penetration.^121^ The ceramic with low porosity in the surface layer and high porosity in the deeper layers mimicked the real enamel and dentin with a gradient of mechanical and optical properties throughout the prostheses when interlocking to polymer. In terms of the scaffold morphology, Algharaibeh took its cue from the pearly substance on the inner surface of the shell, forming an anisotropic laminar arrangement and intertwined inorganic-organic microstructure. It had a more tortuous crack propagation path when cracks occurred, so the mosaic structure of this dental material exhibited high FS, good fracture resistance, and excellent damage tolerance compared to commercial Vita Enamic.^119^

#### Porous nanoclusters

**Fig. 5:**
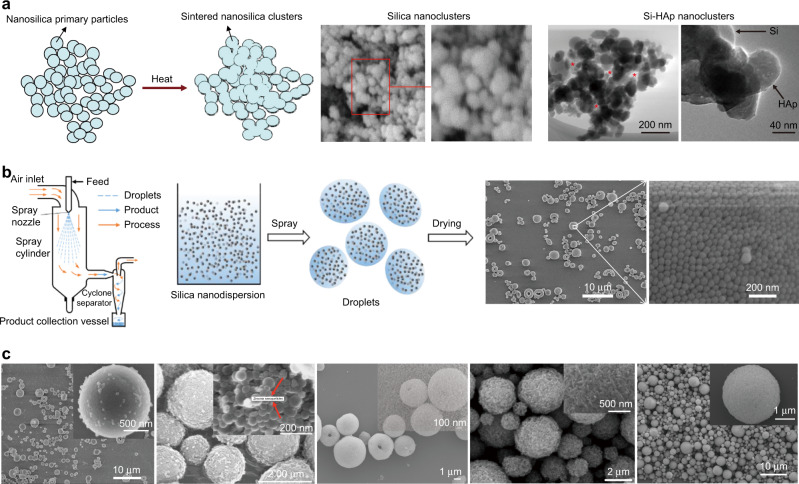
Porous nanoclusters. **a** Schematic diagram of sintering to produce porous nanoclusters, SEM images of sintered silica nanoclusters^124^ and Si-HAp nanoclusters^98^ **b** Schematic^129^ and SEM images of SCNCs synthesized by spray drying method and the mechanical properties of DRCs filled with calcined/uncalcined nanoclusters.^130^ **c** SEM image of SiO_2_-ZnO,^133^ SiO_2_-ZrO_2_,^131^ CaF_2_-SiO_2_,^134^ hydroxyapatite^132^, and SiO_2_-ZrO_2_-ZnO^135^ porous nanoclusters synthesized by spray drying method. Figures adapted with permission from refs. ^98,129–135^

Combined with the advantages of the nanofiller and the micromechanical interlocking structure, 3 M corporation proposed using the nanoclusters as an inorganic component in DRCs in 2003.^122^ These nanoclusters were formed by calcining nano-sized particles in a “bottom-up” method (Fig. 5a), which were not completely dense and has surface pore and interconnected pore structures. These pores became the micromechanical interlocking sites between the filler and resin matrix, providing morphological and positional adaptability during the stressing process. Results demonstrated that these nanoclusters endowed the composites with stronger FS and CS compared to other commercial DRCs. Meanwhile, the nanoparticles improved the polish properties of the composite, which could decrease the adhesion of plaque. Curtis et al. further measured the fracture and cyclic stress loading of this composite in the dry and wet environments, founding that the irregular pores in the nanoclusters allowed the penetration of the resin matrix, which was conducive to forming the filler/resin interface micromechanical interlocking and enhanced interfacial adhesion.^123^ In addition, the nanoclusters can absorb and dissipate the crack stresses according to collapsing into pre-existing cluster pores or losing fragments from the main cluster structure, thus reinforcing the composite.

Atai et al. constructed the silica nanoclusters with suitable porous structure and surface area by controlling the temperature, providing conditions for the formation of micromechanical interlocking structure and the sites for coupling agent action. The physical and chemical enhancement at the interface allowed this material to exhibit higher FS, elastic modulus, and fracture toughness than commercial DRCs.^124^ Reis et al. fabricated Si-HAp porous nanoclusters by co-sintering silica and hydroxyapatite nanoparticles, where the porous is beneficial to constructing the micromechanical interlocking structure or storage fluorion, therefore proving the excellent mechanical properties or the remineralization potential (Fig. 5a).^98^ It is worth mentioning that the fluorine was obtained by immersing the nanoclusters in NaF solution, and the deposited NaF nanoparticles would occupy the pore structure, which is not conducive to the penetration of the resin matrix, thus the composites filled with fluorine-containing porous nanoclusters did not show enhancement of mechanical properties.

However, the porosity and the stacking density of nanoclusters constructed by sintering are difficult to control precisely, decreasing the property stability of DRCs. In addition, the hydroxyl groups density on the nanoparticle surface would decrease during the high-temperature process, which is not beneficial to the modification of coupling agents as well as the mechanical properties. To better control the morphology and pore size of clusters and simplify the reaction conditions, some other synthesis methods, such as covalent coupling,^125,126^ solvent evaporation,^127,128^ and spray drying^129–135^ have been studied.

Covalent coupling method mainly refers to forming the agglomeration by a coupling reaction between amino and epoxy-functionalized nanoparticles, which can be carried out at room temperature.^125^ The size distribution of the nanoclusters can be controlled well with this method, thus improving their wear polishability and polymerization shrinkage properties.^126^ Rodríguez et al. obtained the MPS functionalized silica nanoclusters by solvent evaporation without the high-temperature treatment. The result demonstrated that in addition to the influence of the pores of the clusters, the overall shape and the functionalized modification of the clusters have an obvious effect on the mechanical properties of DRCs, where the porous with 350 nm nanoclusters with spherically shaped could dissipate tension more effectively than irregularly shaped clusters, and the MPS or OTMS modification can affect the connection between the inorganic clusters and the resin matrix.^128^

Compared with the above-mentioned methods, the spray drying method has also been introduced into the synthesis of nanoclusters used in DRCs in recent years. This method can produce nanoclusters with regular morphology and good dispersion, and it is simple, efficient, reproducible, and has the potential for mass production (Fig. 5b).^129^ Yang et al. synthesized the regular porous silica colloidal nanoparticle clusters (SCNCs) with the size of 1–3 μm by adjusting the parameters, and used them in DRCs. By comparison with microfillers of the same size, it was verified that the porous structure of this nanocluster can form a strong filler/resin micromechanical interlocking, improving the stress dissipation of DRCs. To prevent the clusters from decomposing at high loadings, the above nanocluster was further heat-treated at a relatively low temperature, which made the clusters more compact and stable to maintain the excellent properties of the porous nanoclusters.^131^ The result showed that the 500 °C post-treatment obtained stronger internal structure and higher filling content, showing significant improvements in FS (14%), bending modulus (29%), and hardness (52%) compared to the above nanocluster. And using calcined and uncalcined nanoclusters together can take full advantage of micro-nano combination and micromechanical interlocking, allowing the properties of these DRCs to be comparably to the commercial Filtek^TM^ Z350XT.

In addition to the synthesis of silica nanoparticle clusters, the spray drying method has been extended to produce multifunctional high-performance nanoclusters, such as SiO_2_-ZnO,^133^ SiO_2_-ZrO_2_,^131^ CaF_2_-SiO_2_,^135^ hydroxyapatite^132^ and SiO_2_-ZrO_2_-ZnO^135^ (Fig. 5c), all of which can be used to construct high-quality DRCs. In addition, the clusters still presented the spherical tightly packed structure and porous structure although introduce different components, which did not weaken the filler/resin interfacial micromechanical interlocking and could deflect or dissipate the cracks extension. Among all the above nanoclusters, the addition of flake ZnO nanoparticles led to a slight increase in particle size and pore size, which is helpful to promote the penetration of organic resin matrix into the porous clusters. Therefore, the ZnO nanoclusters can enhance the filler/resin interlocking network and endow excellent antibacterial activity of the DRCs, which showed higher FM and hardness over SiO_2_ clusters.^133^ Besides, the preparative SiO_2_-ZrO_2_-ZnO nanoclusters by a three-fluid nozzle effectively avoided the aggregation of particles with opposite potentials during the spray drying process, and the manufactured composites showed excellent mechanical properties, X-ray opacity, and antimicrobial activity.^135^ The design of these functional fillers extends the concept of the combination of structure and function, to obtain biological functions while maintaining and even further enhancing the original properties of corresponding DRCs.

In addition, aerogels are an emerging porous material. The stacked nanoparticles in aerogels are filled with nanoscale open interconnected pore channels. István et al. demonstrated that the pores of silica aerogels (SiA) could be saturated by medical polymers to construct novel bone repair materials, and subsequently, silica aerogels were used as fillers to design DRCs.^136^ Alireza compared the effect of silanized and non-silanized SiA on the mechanical properties of DRCs, demonstrating that 89% of the tensile modulus and 85% of the tensile strength of DRCs came from non-chemical interactions (micromechanical and secondary interactions). While, the fracture toughness did not depend on covalent interactions at the filler/resin interface and was mainly controlled by interfacial micromechanical interlocking.^137^ The FS of DRCs increased with increasing SiA content within a certain range, but excessive filling destroyed the overall structural homogeneity and property stability of the material, leading to a decrease in FS. In further, silica aerogel-filled DRCs had an unexpected antibacterial function, probably because the hydrophobic groups on the aerogel surface affected the permeability of microbial cell membranes. In future research, aerogels can be studied together with other micron or nanoscale fillers to further optimize the various properties of DRCs.

### Fillers with three-dimensional whisker morphology structure

Whiskers, which are high-strength single crystals, have a large length-diameter ratio (several to several hundred or more) and almost no structural defects.^138^ The whiskers have been used as fillers for DRCs,^139–141^ and their morphological structure is conducive to enhancing the mechanical properties of the composites because of the crack deflection, crack bending, microcracking, whisker pull-out, whisker bridging during the fracture process.^142^ While, the results demonstrated that their enhancement effect is not obvious because of their reunite in the resin matrix. Based on this, some researchers designed and synthesized three-dimensional (3D) whisker structure to alleviate the agglomeration of particles and improve the reinforcement of composites. Compared with irregular particles and one-dimensional whiskers, the 3D whisker is conducive to embedding into the resin matrix and forming a micromechanical interlocking structure, thus enhancing the interfacial bonding and improving the properties of reinforced composites.^143^

The 3D tetrapod structure zinc oxide particle (T-ZnOw) was discovered by Kitano in 1990,^144^ and it had a special morphological structure to improve the mechanical properties of composites.^145–147^ The tetrapod structure exerts a dissipative energy effect of steric stress transfer while weakening the negative effect of one-dimensional whisker entanglement into balls. What’s more, zinc oxide has significant antimicrobial activity against the main causative agent of caries (Streptococcus mutans),^148^ which is very meaningful to relieve secondary caries. T-ZnOw was used in DRCs as fillers to evaluate the mechanical properties and antibacterial activity,^149^ and the result showed that incorporation of 5% T-ZnOw provided appropriate antibacterial activity and long-term antibacterial effect simultaneously. Wille et al. tested the biaxial bending strength of composites filled with spherical or tetrapod-shaped ZnO at 60% filler loading, respectively, and found that tetrapod-shaped particles could significantly increase the mechanical properties even when no chemical coupling agent is applied.^150^ The result demonstrated that the tetragonal whiskers in different directions were beneficial to forming the strong micromechanical interlocking and displacement limitation within the resin.

**Fig. 6:**
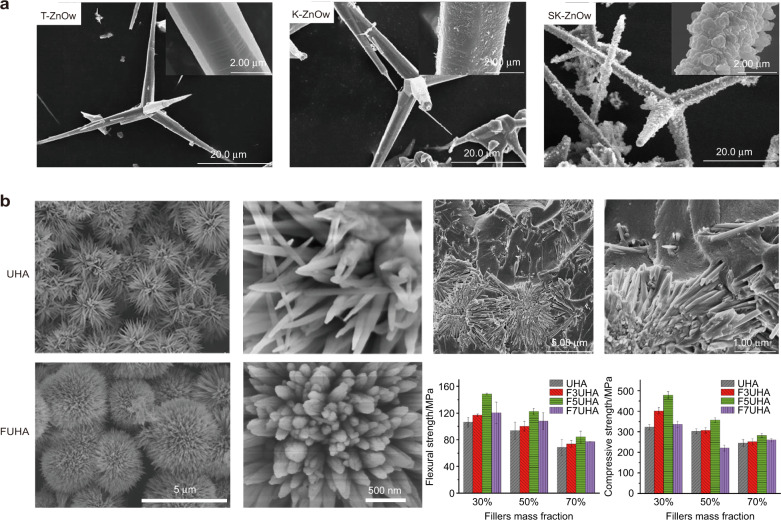
Three-dimensional whisker. **a** SEM images of T-ZnOw, γ-MPS modified T-ZnOw and SiO_2_ hybridized T-ZnOw.^152^ **b** SEM images of UHA and FUHA and their 3D whiskers embedded in resin matrix,^71^ and their effects on the mechanical properties of DRCs.^159^ Figures adapted with permission from refs. ^71,152,159^

However, there were reports that the FS of T-ZnOw-filled composites decreased with the increase of T-ZnOw content.^151^ To solve this problem, the T-ZnOw were silanized by γ-MPS (K-ZnOw) and then hybridized with silica particles on their surface (SK-ZnOw) (Fig. 6a), which combine the physical micromechanical interlocking and chemical coupling bonding. In addition, the silica particles on the whisker surface also improve their surface roughness and reduce the agglomeration of whiskers, resulting in about 50% improvement in FS of DRCs.^152^ This process optimized the filler/resin interface interaction and reduced the polymerization shrinkage (<5%) and water absorption of the composites. In addition, the strong interface bonding could also slow down the diffusion and the loss of antibacterial reactive oxygen species produced by ZnO, resulting in a more stable antibacterial effect of the composite. Therefore, a small amount of SK-ZnOw can finally endow the DRCs with excellent comprehensive properties.

Zhu’s team, inspired by the structure characteristic of sea-urchin and the fact that hydroxyapatite is the main component of tooth,^153^ synthesized the hydroxyapatite particle with 3D urchin-like whiskers (UHA) spherical structure by microwave-assisted irradiation method (Fig. 6b), and then used them as the reinforcement filler in DRCs.^143,154^ The results showed that this urchin-like whiskers filler, presenting a hierarchical spherical structure with a particle size of 2–3 μm, has good dispersion in the resin matrix and the whiskers could embed into the resin matrix in multiple directions, which can form the micromechanical interlocking interface between filler and resin matrix and thus improve the properties of the corresponding composites. This structure combined the enhancement effect of one-dimensional whiskers and interface micromechanical interlocking structure, which therefore more effectively resisted the relative displacement of fillers and optimized the properties of DRCs. However, the synthesis procedure of UHA particle was complex, and its spherical structure and dispersion still needed to be improved. To further optimize the characteristic and synthetic process, the team synthesized the urchin-like serried hydroxyapatite (USHA) particle through a simple and mild hydrothermal reaction with high yield,^71^ and the mechanical properties of USHA-filled DRCs (30% filler loading) was better than commercial DRCs such as Z350XT (3 M) and Spectrum (TPH3). To improve the filler loading and the other properties of USHA-filled DRCs, the filler surface was modified by a silica sol layer through a sol-gel method, which not only roughened the filler surface and therefore facilitate mechanical interlocking, but also increased the grafting rate of the silane coupling agent to improve the filler dispersion and filler loading (50%).^155^ In addition, the hydroxyapatite-filled DRCs presented excellent remineralization properties in oral cavity simulation fluid, which had the potential to alleviate secondary caries.

However, the synthetic hydroxyapatite particle with different morphology structures contained hydroxyl groups, causing the DRCs to absorb water easily in the oral environment, which weakened their mechanical properties and service life.^156,157^ Fluorinated hydroxyapatite particles have been demonstrated to have better biological properties.^158^ Recently, Chen et al. synthesized urchin-like fluorinated hydroxyapatite (FUHA) by simple hydrothermal precipitation and found that this particle, as a filler for DRCs, was more conducive to stress transfer and hindered the polymer migration between the whiskers, which was beneficial to improving the mechanical properties of composites. It should be noted that the mechanical properties of DRCs at high FUHA loading (70%) is lower than 50% loading (FS ~ 135 MPa, CS ~ 340 MPa), which may be due to the large surface area of the 3D whisker fillers and poor filler/resin matrix interface interaction at high filler loading (Fig. 6b).^159^ In addition, the incorporation of fluorine could also reduce the water sorption-solubility properties, enhance the acid resistance, and improve the remineralization and the cytocompatibility of corresponding composites. Therefore, fluorinated hydroxyapatite was a promising particle to improve the mechanical properties and functionality of DRCs.

In addition to the above 3D whisker morphologies particles, other multi-layered whiskers were synthesized by grafting or self-assembling smaller whisker branches onto the backbone. These multi-layered 3D whiskers were also introduced into organic-inorganic composites, which were micromechanically interlocked together by interspersing whisker branches with polymer chains and had better mechanical property enhancement compared to one-dimensional whiskers.^160,161^ Although there are no reports on the application of such structured fillers in DRCs, they should have good prospects for development.

## Conclusion and future insights

**Fig. 7:**
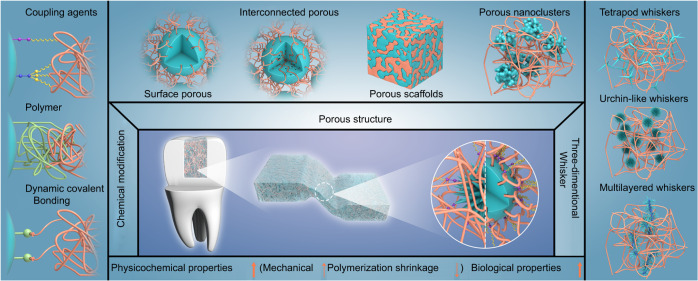
Micromechanical interlocking structure at the filler/resin interface for dental composites

The filler/resin interface interaction has a great influence on the properties of organic/inorganic dental resin composites (DRCs). The excellent interfacial interaction is beneficial to improving the comprehensive properties of DRCs and thus has a positive effect on solving the problem of restoration fracture and secondary caries in service application. Compared with the commonly used chemical bonding between resin and fillers interface, a micromechanical interlocking structure is an easier and more effective method to optimize the filler/resin interface interaction and improve the service life of DRCs. The filler with porous morphology structure can induce the resin matrix to enter the filler pore channel, and the 3D whisker structure is conducive to the filler being embedded in the resin matrix, all of which can form the interpenetrating architecture and thus establish the micromechanical interlocking by the curing process. Although many researchers have introduced the micromechanical interlocking structure into the filler/resin interface and obtained well experimental results in DRCs, there are still some problems that should be studied and refined (Fig. 7). Based on this review, we propose the following research prospects about the micromechanical interlocking structure in DRCs:

1. The porous or three-dimensional structure particles reviewed in this article can be synthesized in large quantities with a simple, mild, and controllable process, and these particles can be used as fillers directly without other chemical modifications. These are important for their clinical applications. However, except for some PICN and nanocluster-loaded DRCs from 3 M corporation, there are few fillers with micromechanical interlocking effects applied to products, most of them are still in laboratory studies. Therefore, it is necessary to systematically research the effects of these fillers on the comprehensive properties of filled DRCs, and explore their clinical application prospects.
2. The morphology structure of porous fillers has a significant effect on the filler/resin interface micromechanical interlocking, while the recent research on porous fillers mainly focused on silica-based particles, which requires us to study more porous fillers with enhancement and functionality, such as alumina, zirconia, hydroxyapatite, and so on, giving further improvement on the properties of DRCs.
3. Most of the current studies only point out that filler/resin interface micromechanical interlocking can strengthen DRCs, while the evaluation of how specific morphological scales such as pore size, porosity, size, and density of three-dimensional whiskers affect the strengthening of DRCs is still unclear. Therefore, there is a need for conditioning and testing to clarify the optimal morphological structure design for the inorganic phase of dental restorative materials and to explain the optimization mechanism.
4. The current studies only give the result that filler/resin interface micromechanical interlocking can effectively improve the mechanical properties, polymerization shrinkage, and cytocompatibility of DRCs, without systematically testing and analyzing the physicochemical properties and biosecurity, such as the properties stability in oral environmental, clinical technical sensitivity, esthetics, and so on. In addition, the interface change should be studied by real-time monitoring to better understand the enhancement mechanism of micromechanical interlocking on DRCs.
5. As for the morphology structure of porous filler or three-dimensional whisker filler, the optimum filling loading is lower than the current commercial DRCs due to their large surface area, and the higher loading is often not easy to infiltrate the three-dimensional structure with the matrix to achieve the best performance. This demands us to construct multimodal fillers with novel compositions based on a micromechanical interlocking structure to improve the enhancement and functional performance more effectively, and finally build the DRCs with high strength, antibacterial, remineralization, radiopaque, and esthetic in one.

## Supplementary information

Read-me of Supplementary information

Supplementary information
