# Supplementary material for: Micromechanical interlocking structure at the filler/resin interface for dental composites: a review
Source: Int J Oral Sci. 2023 May 31;15:21. doi: 10.1038/s41368-023-00226-3 (PMC10232436; doi:10.1038/s41368-023-00226-3)
Supplement: Supplementary file 1 — Read-me of Supplementary information [file 41368_2023_226_MOESM1_ESM.docx]

**Read-me of Supplementary information**

The Supplementary information for this paper includes an image and a table, both in a word document called "Supplementary information".

Fig. S1 describes the development of dental resin composites.

Table S1 summarizes the micromechanical interlocking structure of the filler/resin interface in DRCs.
