# Supplementary material for: Micromechanical interlocking structure at the filler/resin interface for dental composites: a review
Source: Int J Oral Sci. 2023 May 31;15:21. doi: 10.1038/s41368-023-00226-3 (PMC10232436; doi:10.1038/s41368-023-00226-3)
Supplement: Supplementary file 2 — Supplementary information [file 41368_2023_226_MOESM2_ESM.docx]

***Supplementary Information:***

**Micromechanical interlocking structure at the filler/resin interface for dental composites: A review**

Shuning Zhang^1^, Xiao Wang^1^, Jiawei Yang, Hongyan Chen*, Xinquan Jiang*

Department of Prosthodontics, Shanghai Ninth People’s Hospital, Shanghai Jiao Tong University School of Medicine; College of Stomatology, Shanghai Jiao Tong University; Shanghai Engineering Research Center of Advanced Dental Technology and Materials; National Center for Stomatology; National Clinical Research Center for Oral Diseases; Shanghai Key Laboratory of Stomatology; Shanghai Research Institute of Stomatology. No. 639 Zhizaoju Road, Shanghai, 200011, China

*Corresponding author.

E-mail addresses: hychen_1201@163.com, xinquanj@aliyun.com (X. Jiang).

^1^ These authors contributed equally to this work


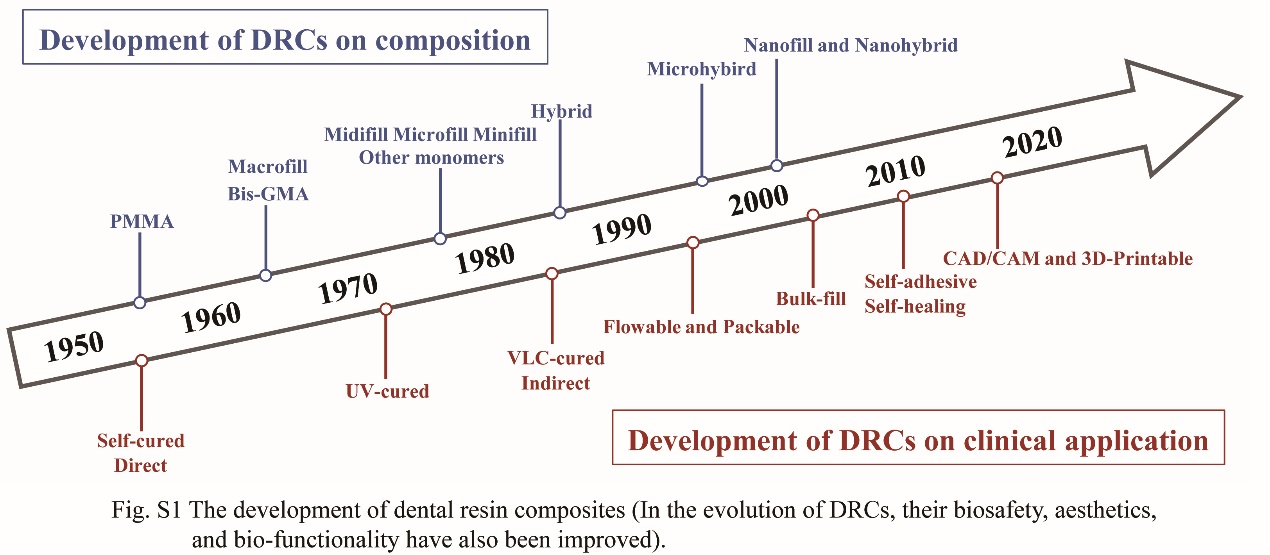


**Fig. S1.** The development of dental resin composites (In the evolution of DRCs, their biosafety, aesthetics, and bio-functionality have also been improved)

**Table S1** Summary the micromechanical interlocking structures at the filler/resin interface in DRCs

| **Table S1** Summary the micromechanical interlocking structures at the filler/resin interface in DRCs | | | | |
| --- | --- | --- | --- | --- |
| Filler Structure | Filler Name | Filler Content (wt%) | Effects on DRCs | Ref. |
| **Porous fillers** | | | | |
| Surface porous | ▪ HF-catalyzed silanized silica gel | 42 | ·Excellent wear resistance | ^75^ |
|  | ▪ HF-etched IPS Empress silanized particles | 77 | ·Enhancement mechanical properties (DTS~39.8 MPa, FS~78.5 MPa, FM~12.8 GPa) | ^76^ |
|  | ▪ Porous silanized fluorapatite glass-ceramic particles | 75 | ·Excellent wear resistance  ·Enhancement FS (~75.5 MPa) | ^77^  ^78^ |
|  | ▪ Wrinkled silanized mesoporous silica (WMS) | 35 | ·Enhancement mechanical properties (FS~115 MPa, FM~6.2 GPa, CS~225 MPa, VH~35 HV) | ^86^ |
|  | ▪ Rough silanized core-shell SiO_2_ | 67 | ·Enhancement mechanical properties  (FS~120 MPa, FM~5 GPa, CS~330 MPa)  ·Excellent cytocompatibility | ^87^ |
|  | ▪ Zn doped silanized mesoporous silica nanoparticles (Zn-MSNs) | 15 | ·Enhancement mechanical properties (FS~131.26 MPa, FM~3.16 GPa, CS~442.5 MPa, VH~36.2 HV)  ·Excellent cytocompatibility  ·Antibacterial rate~100%  ·Decline of polymerization shrinkage (~7.25%) | ^88^ |
|  | ▪ Silanized santa Barbara amorphous 15 (SBA-15) | 39 | ·Excellent cytocompatibility | ^89^ |
|  | ▪ Chlorhexidine-encapsulated MSNs (CHX@MSN) and Glass | CHX@MSN:glass =7.5:62.5 | ·Enhancement mechanical properties  (FS~90 MPa, FM~7 GPa)  ·Excellent cytocompatibility  ·Continuous antimicrobial activity | ^98^ |
|  | ▪ Silanized ZnO@m-SiO_2_ and SiO_2_ | ZnO@m-SiO_2_: SiO_2_ = 7:63 | ·Enhancement mechanical properties  (FS~208.6 MPa, FM~6.7 GPa, CS~312.4 MPa)  ·Antibacterial rate~99.9%  ·Polymerization shrinkage reduction (~2.97%) | ^90^ |
|  | ▪ Ca-Doped mesoporous SiO_2_ | 20 | ·Enhancement mechanical properties  Surface porous  (FS~109.6 MPa, FM~3.01 GPa, CS~460 MPa, Knoop Hardness~24.38)  ·Induction apatite remineralization  ·Drug delivery and drug slow release | ^102^ |
|  | ▪ Ca doped silanized wrinkled porous silica (Ca-WPS) and silanized silica | Ca-WPS: silica = 10:50 | ·Enhancement mechanical properties (FS~132.42 Pa, FM~8.82 Pa, CS~294.09 Pa, VH~63 V)  ·Induction apatite remineralization | ^103^ |
| Interconnected porous | ▪ Mesoporous silica and spherical silica | Mesoporous silica=40 | ·CM~5.7 GPa (resistance to hydrolytic de-bonding), CS~191 MPa, FM~6.7 GPa | ^85^ |
|  |  | Mesoporous silica: spherical silica = 21:49 | ·Enhancement mechanical properties  (CM~8.1 GPa, CS~224 MPa, FM~10.3 GPa) | ^73^ |
|  | ▪ Dendritic porous silica (DPS) | 36 | ·Enhancement mechanical properties  (FS~123 MPa, FM~6.5 GPa, CS~450 MPa，Work of fracture~2.1 KJ/m^2^)  ·Excellent cytocompatibility | ^104^ |
|  | ▪ Anodic porous alumina (APA) | 50 | ·Storage modulus~5 GPa (In oral masticatory environment and Anti-aging)  ·FM~13.2 GPa (Anti-aging)  ·Drug delivery carrier | ^105^  ^107^ |
|  | ▪ Functionalized nanostructured titania tubes (TNT) | 1 in cement | ·Enhancement mechanical properties (FS~90.73 MPa, FM~3.81 GPa, K_IC_ ~1.42 MPa·m^1/2^)  ·Excellent cytocompatibility | ^108^ |
|  |  | 3 in flowable restorative | ·Enhancement mechanical properties (Dynamic elastic moduli~16.8 GPa, K_IC_ ~1.18 MPa·m^1/2^)  ·Excellent cytocompatibility | ^110^ |
|  |  | TNT: SiO_2_=3:60 | ·Enhancement mechanical properties  (FS~128.9 MPa, FM~5.7 GPa, CS~188.4 MPa)  ·Wear resistant  ·Reduced water absorption | ^111^ |
| Porous scaffolds | ▪ Zirconia-reinforced ceramic network | 86 | ·Enhancement mechanical properties  (FS~161.2 MPa, FM~23.23 GPa, CS~560.4 MPa, DTS~43.5 GPa, VH~1.86 GPa) | ^113^ |
|  | ▪ Nanoscale porous silica block | 46 | ·VH~54 HV (comparable to dentin), E ~7 GPa | ^114^ |
|  |  | 86 | ·VH~397 HV (comparable to enamel),  E ~28GPa |  |
|  | ▪ Nanoscale porous silanized silica block | 71.2-89.6 | ·Enhancement mechanical properties (FS~107-154 MPa, FM~13-22.2 GPa comparable to dentin, VH~200.8-299.2 HV) | ^115^ |
|  | ▪ Porous silanized ZrO_2_ scaffold | 70 | ·Enhancement mechanical properties  (FS~265.5 MPa, Hardness~3.3 GPa) | ^116^ |
|  | ▪ Porous silanized silicon nitride scaffold  Porous scaffolds | 60 | ·Enhancement mechanical properties  (FS~385.3 MPa, VM~2.4 GPa, E ~59.3 GPa)  ·Excellent cytocompatibility | ^117^ |
|  | ▪ Graded Alumina scaffold | / | ·Mechanical properties similar to dentin (E~6.5-9.1GPa, FS~183.1-213.1MPa, CS~140.6-22.4MPa from bottom to upper layers) | ^119^ |
|  | ▪ Silanized graded glass-ceramic porous network | 72.4-82.4 vol% | ·Mechanical properties (FS~175.8-372.7 MPa, E~20-41.9 GPa, VH~3.8-0.4 GPa from enamel to dentin zone)  ·Higher flexural Load Energy  ·Glossy improvement | ^120^ |
|  | ▪ Brick-and-mortar microstructure silanized Al_2_O_3_/MgO ceramic | 70 vol% | ·Enhancement Mechanical properties and biomimetic (E~57.75-61.22 GPa, FS~135.08-145.77 MPa, VM~3.07-3.36 GPa, K_IC_ ~2.54 MPa·m^1/2^) | ^118^ |
| Porous nanoclusters | ▪ Silanized sintered ZrO_2_/SiO_2_ nanocluster and SiO_2_ nanoparticle | SiO_2_: ZrO_2_/SiO_2_= 8:71 | ·Enhancement mechanical properties (DTS~80.7 MPa, FS~153.1 MPa, CS~426.2 MPa, K_IC_ ~1.2 MPa·m^1/2^)  ·Excellent gloss retention | ^121^ |
|  | ▪ Silanized sintered SiO_2_ nanocluster and SiO_2_ nanoparticle | Nanoparticle: Nanocluster= 40:30 | ·Enhancement mechanical properties (DTS~87.6MPa, FS~177.1MPa, CS~458.6MPa, K_IC_ ~1.3MPa·m^1/2^)  ·Excellent wear resistance and gloss retention | ^122^ |
|  |  | Nanocluster =70% | ·Enhancement mechanical properties  (DTS~33 MPa, FS~105 MPa, FM~7.5 GPa, K_IC_ ~1.65 MPa·m^1/2^)  ·Smoother surface | ^123^ |
|  | ▪ Sintered Si-HAp nanoporous particle | 60 | ·Induction of apatite remineralization  (Nano-pores store NaF)  ·Mechanical properties enhancement  (FS~80.2 MPa but decreased after storage NaF) | ^96^ |
|  | ▪ Silanized SiO_2_ nanoparticle and nanocluster | Nanoparticles: Nanoclusters =50:20 | ·Improvement wear behavior, conversion rate, and polymerization shrinkage  ·Enhancement mechanical properties  (FS~104.8 MPa, FM~6.2 GPa, CS~205.8 MPa)  ·Excellent cytocompatibility | ^125^ |
|  | ▪ Silanized SiO_2_ nanocluster from solvent evaporation | 30 | ·Nanoclusters shape affect flow behavior  ·Increased CS | ^126^ |
|  | ▪ Silanized SiO_2_ colloidal nanoparticle clusters (SCNCs) from spray drying and Calcined SCNCs (CSCNCs)  Porous nanoclusters | SCNCs=70 | ·Enhancement mechanical properties (FS~125 MPa, FM~7 GPa, CS~370 MPa，HM~45 HV) | ^127^ |
|  |  | CSCNCs: SCNCs=60:10 | ·Enhancement mechanical properties  (FS~143 MPa, FM~8.91 GPa, CS~370 MPa，HV~70.7 HK) | ^128^ |
|  | ▪ Silanized SiO_2_-ZrO_2_ nanoclusters | 30 | ·Higher roughness and constant nanohardness compared to silica nanopaticles | ^129^ |
|  | ▪ Silanized Micro-sized clusters of hydroxyapatite nanorods | 50 | ·Enhancement mechanical properties (FS~119.6 MPa, FM~5.6 GPa, CS~414.9 MPa)  ·Induction of apatite remineralization | ^130^ |
|  | ▪ Silanized SiO_2_-ZnO complex clusters | 70 | ·Enhancement mechanical properties  (FS~120 MPa, FM~8.1 GPa, CS~382.8 MPa，VH~53.2 HV)  ·Antibacterial rate~99% | ^131^ |
|  | ▪ Silanized CaF_2_/SiO_2_ nanoclusters | 55 | ·Enhancement mechanical properties (FS~127.8 MPa, FM~7.3 GPa, CS~422.4 MPa, VH~69.3 HV)  ·Antibacterial rate~93% | ^132^ |
|  | ▪ Silanized SiO_2_-ZrO_2_-ZnO nanoparticle clusters | 70 | ·Enhancement mechanical properties (FS~130MPa, FM~8.5GPa, CS~.365MPa，VH~61.7HV)  ·Antibacterial rate~99.9% | ^133^ |
|  | ▪ Mesoporous silica aerogel (SiA) | 12.5 | ·Enhancement mechanical properties (FS~62.546 MPa, Shore Hardness ~100 A)  ·Low water absorption and dissolution  ·Excellent antibacterial property | ^136^ |
|  | ▪ Silanized SiA | 15 | ·Enhancement mechanical properties（FM~1.7 GPa, FS~95 MPa, K_IC_ ~0.65 MPa·m^1/2^） | ^135^ |
| **Three-dimensional Whisker Structure** | | | | |
| Tetrapod whiskers | ▪ Tetrapod-like zinc oxide whisker (T-ZnOw) | T-ZnOw =60 | ·Enhancement mechanical properties  (FS~ 111.2 MPa) | ^148^ |
|  | ▪ Tetrapod-like zinc oxide whisker (T-ZnOw) and SiO_2_ | T-ZnOw: SiO_2_=5:67.6 | ·Enhancement mechanical properties (FS~134.6 MPa, CS~200.9 MPa，DTS~56.8 MPa)  ·Antibacterial rate~94.2% | ^147^ |
|  | ▪ Silanized SiO_2_ particles hybridized ZnOw, (SK-ZnOw) | 20 | ·Stable antibacterial activity (>90%)  ·Low polymerization shrinkage (~5%)  ·Enhancement mechanical properties (FS~83 MPa, FM~2.3 GPa, VH~56 HV) | ^150^ |
| Urchin-like whiskers | ▪ Silanized urchin-like hydroxyapatite (UHA) and SiO_2_ | UHA=20 | ·Enhancement mechanical properties  (FS~118 MPa, FM~3.8 GPa, CS~388.7 MPa, VH~30 HV, DTS~46.2 MPa) | ^154^ |
|  |  | UHA: SiO_2_=10:50 | ·Enhancement mechanical properties  (FS~125 MPa, FM~10 GPa, CS~425 MPa) |  |
|  | ▪ Silanized urchin-like serried hydroxyapatite (USHA) | 30 | ·Enhancement mechanical properties (FS~118 MPa, FM~4 GPa, CS~460 MPa, VH~27 HV) | ^155^ |
|  | ▪ Silanized sol-gel layer coating USHA (TUSHA) | 50 | ·Enhancement mechanical properties  (FS~125 MPa, FM~6 GPa, CS~350 MPa)  ·Induction of apatite remineralization | ^152^ |
|  | ▪ Silanized fluorinated urchin-like serried hydroxyapatite (FnUHA) | 50 | ·Enhancement mechanical properties  (FS~135 MPa, CS~340 MPa)  ·Excellent cytocompatibility  ·Induction of apatite remineralization | ^159^ |
| DTS: diametral tensile strength; FS: flexural strength; FM: flexural modulus; CS: compressive strength; VH: Vickers hardness; K_IC_: fracture toughness; E: Elastic modulus; CM: Compression modulus | | | | |
